# The value of early root development traits in breeding programs for biomass yield in perennial ryegrass (*Lolium perenne* L.)

**DOI:** 10.1007/s00122-024-04797-5

**Published:** 2025-01-21

**Authors:** M. Malinowska, P. S. Kristensen, B. Nielsen, D. Fè, A. K. Ruud, I. Lenk, M. Greve, T. Asp

**Affiliations:** 1 Center for Quantitative Genetics and Genomics, Aarhus University, Slagelse, Denmark; 2 Research Division, DLF Seeds A/S, Store Heddinge, Denmark; 3 Department of Plant Sciences, Norwegian University of Life Sciences, Ås, Norway

## Abstract

**Key message:**

**Early root traits, particularly total root length, are heritable and show positive genetic correlations with biomass yield in perennial ryegrass; incorporating them into breeding programs can enhance genetic gain.**

**Abstract:**

Perennial ryegrass (*Lolium perenne* L.) is an important forage grass widely used in pastures and lawns, valued for its high nutritive value and environmental benefits. Despite its importance, genetic improvements in biomass yield have been slow, mainly due to its outbreeding nature and the challenges of improving multiple traits simultaneously. This study aims to assess the potential advantages of including early root traits in the perennial ryegrass breeding process. Root traits, including total root length (TRL) and root angle (RA) were phenotyped in a greenhouse using rhizoboxes, and genetic correlations with field yield were estimated across three European locations over two years. Bivariate models estimated significant genetic correlations of 0.40 (SE = 0.14) between TRL and field yield, and a weak but positive correlation to RA of 0.15 (SE = 0.14). Heritability estimates were 0.36 for TRL, 0.39 for RA, and 0.31 for field yield across locations. Incorporating root trait data into selection criteria can improve the efficiency of breeding programs, potentially increasing genetic gain by approximately 10%. This results highlight the potential of early root traits to refine selection criteria in perennial ryegrass breeding programs, contributing to higher yield and efficiency.

**Supplementary Information:**

The online version contains supplementary material available at 10.1007/s00122-024-04797-5.

## Introduction

Maximising genetic gain in biomass yield is a key objective in breeding programs of perennial ryegrass (*Lolium perenne* L.), a forage grass widely used in pastures and lawns. As a component of sustainable agricultural systems, perennial ryegrass offers natural and cost-effective feed options with high nutritive value for dairy and livestock sectors, promotes soil and water resource conservation, reduces erosion, and contributes to biodiversity within agricultural landscapes (Humphreys 2005; Katuwal et al. 2013; Pilgrim et al. 2010). Despite its agronomic importance, progress in breeding has been modest, with annual improvements in yield ranging between 0.2% and 0.6% under grazing conditions (Wilkins and Humphreys 2003; Conaghan et al. 2008; McDonagh et al. 2016) The slow pace of genetic gain in perennial ryegrass breeding is attributed to several inherent complexities, including its predominantly outbreeding nature, the weak correlation between selection based on individual plants and performance in swards, lengthy breeding cycles, and the complexity of simultaneously improving multiple economic traits (Humphreys 1997; Fè et al. 2015b).

Yield, defined as the total biomass or harvestable output, is a quantitative trait shaped by a complex interplay of genetic, environmental, and management factors (Voss-Fels et al. 2019). Heritability estimates for yield in perennial ryegrass typically range between 0.3 and 0.6, indicating moderate to high genetic influence (Devey et al. 1989; Conaghan et al. 2008; Fè et al. 2015a). However, improving yield through traditional methods remains challenging. Field trials, necessary for accurate yield measurement, are costly, time-consuming and limited in their ability to handle large numbers of replicates, and the need for diverse environmental testing to account for genotype-by environment-interactions further complicates the process (Wilkins and Humphreys 2003). Additionally, genotype-by-environment interactions significantly affect yield, complicating the selection of genotypes with consistently superior performance across varying environments (Conaghan et al. 2008).

To enhance breeding efficiency, it might be beneficial to identify correlated traits to increase predictability of yield in the early stages of breeding programs. Root development has emerged as a trait of interest in plant breeding aimed at improving yield and performance across diverse environments (Hammer et al. 2009; Nacry et al. 2013; Wang et al. 2016). Efficient early root development can improve plant's ability to establish, persist, and perform well in different environmental conditions (Lynch 2013, 2007; White 2019). This is because roots play an important role in nutrient and water uptake, anchorage, mechanical support, storage, and overall plant health (Herder et al. 2010; Smith and Smet 2012). Research on crops like wheat, rice, maize, soybean and legumes have shown the significant link between root development and plant growth (Uga et al. 2013; Sandhu et al. 2017; Adeleke et al. 2020; Müller et al. 2020; Chen et al. 2021). Recognizing the importance of early root development and integrating its evaluation into breeding programs can contribute to higher yields and better forage quality, as well as improved adaptability to nutrient-poor and water-limited environments (Herder et al. 2010). In perennial ryegrass, the potential of early root traits as proxies for field yield has not been thoroughly investigated. Integrating root traits measured under glasshouse environment offers a potentially cost-effective and reliable method for predicting field performance, addressing the limitation of traditional field-based selection.

Multivariate models are valuable for integrating multiple correlated traits into the breeding process (Thompson and Mayer 1986; Jia et al. 2012; Arojju et al. 2020). The use of multivariate models allows for estimation of the genetic correlation between traits and potentially improve the accuracy of genomic estimated breeding values predictions, thus enhancing selection accuracy and genetic gain (Jia et al. 2012; Mrode 2014; Rutkoski et al. 2016; Hayes et al. 2017). In our study we used bivariate models to assess the genetic correlations between early root development traits and biomass yield in perennial ryegrass. Our focus was on implementing early root traits, which can be phenotyped under greenhouse conditions, to act as indicators for field yield. Incorporating these traits into selection criteria, aims to improve the breeding process by increasing the accuracy of the predictive yield outcomes and enhancing the overall genetic gain (Fernandes et al. 2018; Mrode 2014). This approach could address one of the practical challenges of field-based selection, replacing one replicate with cost-effective and scalable alternative through early root phenotyping.

We hypothesize that early root development traits of young seedlings can act as proxies for field yield across multiple years and locations. Additionally, we propose that incorporating these traits into selection criteria will increase the genetic gain in a commercial perennial ryegrass breeding program. By estimating the genetic correlation between early root development traits and yield in field experiments in a commercial breeding program, we aim to assess the potential advantages of incorporating early root traits into the selection process.

## Materials and methods

### Plant material and genotyping

In total 239 families of diploid forage type perennial ryegrass were provided by DLF Seeds (Store Heddinge, Denmark). Of these, 235 were F_2_ full-sib families from a commercial breeding program, while the remaining four were commercial varieties (Abosan, Bovini, Fabiola, and Goyave). F_2_ families originated from pair-crosses between single plants (self-pollination avoided due to self-incompatibility), after which seeds from both plants were pooled to form an F_1_ family and then multiplied in isolated plots (random mating occurring within each family from F_1_ to F_2_).

Sequence data was generated using the Genotyping-by-Sequencing (GBS) approach, following the protocols described by Byrne et al. (Byrne et al. 2013) and Elshire et al. (Elshire et al. 2011). For genotyping F_2_ families and commercial varieties, a pooled sample of approximately 100 seedlings per family grown on Rockwool blocks was utilized. Genomic DNA extracted from the pooled samples was digested using the ApeKI (5-bp recognition site) and PstI (6-bp recognition site) restriction enzymes and subsequently sequenced on an Illumina HiSeq4000 platform. The resulting sequencing data were aligned to the reference genome of *L. perenne* (Nagy et al. 2022). Genetic variants were called using GATK 4.2.6.1 and filtered based on a quality score of 30, a missing rate above 0.5, and a minor allele frequency (MAF) below 0.01. After quality filtering a total of 123,231 SNPs were retained for further analyses.

### Experimental setup and growth conditions

Plants were grown in custom-made plastic rhizoboxes (20 cm × 2.5 cm × 37 cm) with a transparent plexiglass front plate. The plexiglass was covered with black plastic foil to prevent algae growth. Rhizoboxes were angled at 60° relative to the horizontal plane, with the plexiglass plate positioned beneath them, promoting root growth along the visible side (Supplemental Figure 1). Each rhizobox was filled with 1.8 L of substrate (a mix of peat and local topsoil) and manually supplemented with 300 ml of water. Three seeds were sown in each box at equal distances between each other, with three boxes (replicates) used for each family (Supplemental Figure 1b).

An incomplete split-plot design was used to allow phenotyping of 239 families in triplicate. To accommodate the limited number of available rhizoboxes (180), the phenotyping process was divided into four blocks, with each block containing 179 families. The commercial variety Abosan consistently served as the control line, occupying the 180th entry in each block to ensure consistency.

The experiment was conducted over three months, from August to December, within a single experimental greenhouse compartment (Research Center Flakkebjerg, Aarhus University, Denmark). The greenhouse conditions were controlled with additional heating and LED artificial lighting (16/8 h and 20/14 °C day/night). After sowing, all families in each block were allowed to grow for 21 days before data collection.

### Root imaging and analysis

Non-destructive root phenotyping was conducted 21 days after sowing (DAS). A Sony RX0 camera was used in a light box to ensure consistent illumination, capturing high-resolution visual spectrum images (3200 × 4272 pixels) for each rhizobox. A total of 718 images were obtained, with three pictures per family, and up to three plants per box.

Images were analysed to extract relevant root traits. Prior to analysis, the images were cropped (1750 × 3100 pixels) to remove the background. Image segmentation was performed using RootPainter (Smith et al. 2020), a software that utilizes a GPU server and convolutional neural network-based deep learning models. The training dataset consisted of 300 root images measuring 700 × 700 pixels. Training and annotation were completed within 210 min, following the protocol established by Smith et al. (2020). The resulting model was used for root segmentation and total root length (TRL) extraction from each image. The software generated TRL values, expressed as the total number of root pixels per box, capturing the cumulative root length for all three plants grown in each rhizobox.

RootPainter and SeminalRootAngle tool (Smith et al. 2024) were used to record the root angle (RA) for each seedling in the rhizobox. RootPainter was used to segment the roots and identify the position of each seed within the box. The SeminalRootAngle tool then generated a skeletal structure of the root system by defining a semi-circle around the root origin. This approach included cropping to exclude roots beyond a specific radius. The RA was finally measured as the angle between the two outermost roots relative to the seed’s position.

### Field experiment

The F_2_ families were sown in October 2020 in sward plots at three locations: Store Heddinge Denmark, Stanway, United Kingdom (UK), and Ballycanvan Big, Ireland. The same plants were maintained throughout two consecutive harvest seasons (2021 and 2022). Plot sizes were 12.5 m^2^ in Denmark and 11.5 m^2^ in the other locations, arranged in 14 trials (or blocks), each containing 18 families. In Denmark, trials were sown in two replicates, and families within each trial were arranged in a Latin square design. In UK and Ireland, only one replicate per trial was sown, precluding a Latin square arrangement in those locations. Families within trials were selected to have similar heading date, and plots were randomized within each trial/replicate. The experiment followed specific management practices at each location with families grown for two harvest years. In Denmark conservation management was followed with 4 cuts per year (each leaving 6 cm of grass). In Ireland simulated grazing management resulted in more frequent cuts (leaving 3 cm of grass): 9 times in 2021 and 6 times in 2022. In the UK, conservation management with 4 cuts was followed in 2021, but due to poor growth caused by drought, only 4–5 cuts were harvested in 2022 under simulated grazing management.

### Traits analyzed

Three key traits were analyzed in this study: TRL, RA, and biomass yield. TRL and RA were measured under glasshouse conditions, while biomass yield was recorded in the field across three locations over two consecutive harvest seasons. TRL represents the cumulative root length per rhizobox, derived from the segmented root images. RA measures the angular spread between the outermost roots of each seedling. Both traits were quantified using the image analysis methods detailed in Sect. “Root imaging and analysis”. Biomass yield (kg of green matter per plot) was measured in field trials at three locations with varying management practices (Sect. “Field experiment”). To account for the wide variability in yields across locations and cuts, the data was log-transformed to normalize it and stabilize variance across the dataset.

### Statistical model and methods

Root recording (TRL and RA) in the rhizobox experiment was designed as an incomplete block design of $n_{a} = 239$ families and three replicates in four incomplete blocks denoted, $j = \left\{ {1,2,3,4} \right\}$. Recording of roots were modelled by

1 $$z_{ij} = \alpha_{i} + \varphi_{j} + \varepsilon_{ij}$$

where $z_{ij}$ is the mean root recording of up to three germinated full-sib seeds, $n_{ij} \in \left\{ {1,2,3} \right\}$ in a rhizobox, i.e. in all boxes at least one seed germinated in each replicate for family $i = \left\{ {1,2, \ldots ,n_{a} } \right\}$ sown in rhizobox $ij$ of block $j$, $\varphi_{j}$ is the fixed block effect, $\alpha_{i} \sim N\left( {0,\sigma_{\alpha }^{2} {\varvec{G}}} \right)$ is the family effect, ***G*** is the genomic relationship matrix calculated using a modification of VanRaden’s (VanRaden 2008) method, as adapted by Cericola et al. (Cericola et al. 2018), which incorporates family-level allele frequency information instead of individual genotypes, and $\varepsilon_{ij} \sim N\left( {0,\sigma_{\varepsilon }^{2} } \right)$ is the residual effect.

The field data of yield $y_{ikl\tau }$ was modelled by

2 $$y_{ikl\tau } = \mu_{l\tau } + \beta_{k\tau } + a_{i} + \gamma_{il} + \epsilon_{ikl} + e_{ikl\tau }$$

where $\mu_{l\tau }$ is the fixed yield level at location $l$ and cut $\tau$, $\tau = \left\{ {1,2, \ldots ,n_{l} } \right\}$, $n_{l}$ is the number of cuts over the two years of the experiment at the three location, $l = \left\{ {1,2,3} \right\}$, $\beta_{k\tau }$ is the combined field trial (or block) and cut effects, $\beta_{k\tau } \sim {\text{N}}\left( {0,\sigma_{\beta \tau }^{2} } \right)$ of field trial $j$ = $\left\{ {1,2, \ldots m_{l} } \right\}$, $m_{l} = 14$, $a_{i}$ is the additive genetic family effect, $a_{i} \sim N\left( {0,\sigma_{a}^{2} {\varvec{G}}} \right)$ of family $i$, $\gamma_{il}$ is the combined family-location effect, $\gamma_{il} \sim {\text{N}}\left( {0,\sigma_{a\gamma }^{2} } \right)$ covering additive and nonadditive genetic interaction effects, $\epsilon_{ikl}$ is the random variation between plots $\epsilon_{ikl} \sim N\left( {0,\sigma_{\epsilon}^{2} } \right)$, $e_{ikl\tau }$ is the random residual variance between cuts within plots $e_{ikl\tau } \sim N\left( {0,\sigma_{e}^{2} } \right)$.

The two above models in Eq. (1) and Eq. (2) were combined in a bivariate model where:

3 $$\left( {\begin{array}{*{20}c} {\varvec{\alpha}} \\ {\varvec{a}} \\ \end{array} } \right)\sim N\left( {\begin{array}{*{20}c} 0 \\ 0 \\ \end{array} ,\left[ {\begin{array}{*{20}c} {\sigma_{\alpha }^{2} } & {\sigma_{\alpha a}^{{}} } \\ {\sigma_{\alpha a}^{{}} } & {\sigma_{a}^{2} } \\ \end{array} } \right] \otimes {\varvec{G}}} \right), \left( {\begin{array}{*{20}c} {\varepsilon_{is} } \\ {e_{ijl\tau } } \\ \end{array} } \right)\sim N\left( {\begin{array}{*{20}c} 0 \\ 0 \\ \end{array} ,\begin{array}{*{20}c} {\sigma_{\varepsilon }^{2} } & 0 \\ 0 & {\sigma_{e}^{2} } \\ \end{array} } \right),$$

${\varvec{\alpha}} = \left( {\alpha_{1} , \ldots \alpha_{i} , \ldots } \right)^{{\mathbf{T}}}$ and ${\varvec{a}} = \left( {a_{1} , \ldots a_{i} , \ldots } \right)^{{\mathbf{T}}}$ are vectors of additive genetic family effects of all individuals in ${\varvec{G}}$, and $\sigma_{\alpha }^{2}$, $\sigma_{a}^{2}$ and $\sigma_{\alpha a}^{2}$ are associated genetic variances and co-variances. The remaining random effects, $\beta_{j\tau }$, $\gamma_{il}$, and $\epsilon_{ijl}$ were assumed to be uncorrelated.

For the field data an extended version of Eq. (3) is represented by Eq. (4):

4 $$ \left( {\begin{array}{*{20}c} {\varvec \alpha } \\ {\varvec a_{1} } \\ {\begin{array}{*{20}c} {\varvec a_{2} } \\ {\varvec a_{3} } \\ \end{array} } \\ \end{array} } \right)\sim N\left( {\begin{array}{*{20}c} {\begin{array}{*{20}c} 0 \\ 0 \\ 0 \\ \end{array} } \\ 0 \\ \end{array} ,\left[ {\begin{array}{*{20}c} {\sigma_{\alpha }^{2} } & {\begin{array}{*{20}c} {\sigma_{\alpha a1}^{{}} } & {\sigma_{\alpha a2}^{{}} } & {\sigma_{\alpha a3}^{{}} } \\ \end{array} } \\ {\begin{array}{*{20}c} {\sigma_{\alpha a1}^{{}} } \\ {\sigma_{\alpha a2}^{{}} } \\ {\sigma_{\alpha a3}^{{}} } \\ \end{array} } & {\begin{array}{*{20}c} {\sigma_{a1}^{2} } & {\sigma_{a12}^{{}} } & {\sigma_{a13}^{{}} } \\ {\sigma_{a12}^{{}} } & {\sigma_{a2}^{2} } & {\sigma_{a23}^{{}} } \\ {\sigma_{a13}^{{}} } & {\sigma_{a23}^{{}} } & {\sigma_{a3}^{2} } \\ \end{array} } \\ \end{array} } \right] \otimes \varvec G} \right), \left( {\begin{array}{*{20}c} {\varepsilon_{is} } \\ {e_{ijl\tau } } \\ \end{array} } \right)\sim N\left( {\begin{array}{*{20}c} 0 \\ 0 \\ \end{array} ,\begin{array}{*{20}c} {\sigma_{\varepsilon }^{2} } & 0 \\ 0 & {\sigma_{e}^{2} } \\ \end{array} } \right), $$

where ${\varvec{a}}_{1}$, ${\varvec{a}}_{2}$, and ${\varvec{a}}_{3}$ are additive genetic effects in the three locations associated with the variances and co-variances across all individuals included in ${\varvec{G}}$. The non-additive genetic interaction effects for family-locations were estimated in $\gamma_{il}$.

Let ${\varvec{y}} = \user2{ }\left( {z_{11} ,z_{ij \ldots } ,y_{1111} ,y_{ikl\tau } , \ldots } \right)^{{\text{T}}}$ be a vector of all recording from both traits, i.e. boxes and field experiments, where ${\text{T}}$ represents the transpose of the vector. Let ${\varvec{y}}^{*} = {\varvec{K}}^{{\text{T}}} {\varvec{y}}$ be a linear function of ${\varvec{y}}$ so that ${\varvec{y}}^{*}$ contains none of the fixed effects which are part of ${\varvec{y}}$. Hence the REML estimate of the random parameters in ${\varvec{\theta}}$ is then obtained by the maximum of the log-likelihood function $\ell \left( {{\varvec{y}}^{*} |{\varvec{\theta}}} \right)$, and for the first trait in the bivariate model (Eq. (3)) recorded as mean values in the boxes given by $z_{ij}$ we have:

5 $$\ell \left( {{\mathbf{y}}^{*} |{{\varvec{\uptheta}}}} \right) \approx \mathop \sum \limits_{{{\text{ij}}}}^{{}} {\text{lnf}}\left( {\frac{{{\upvarepsilon }_{{{\text{ij}}}} }}{{{\text{n}}_{{{\text{ij}}}} }}} \right)$$

where $f$ is the Gaussian probability function applied on each of the residuals $\varepsilon_{ij}$ which are weighed according to $1/n_{ij}$ serving as a correction factor to account for varying number of seeds, $n_{ij}$ defined in Eq. (1) to be the number of germinated seeds in a rhizobox $ij$. Narrow sense heritability of average root records in rhizoboxes was calculated as:

6 $$h_{\alpha }^{2} = \frac{{D \sigma_{\alpha }^{2} }}{{D \sigma_{\alpha }^{2} + \frac{{\sigma_{\varepsilon }^{2} }}{{n_{s} *\overline{{n_{ij} }} }}}}$$

where $\sigma_{\alpha }^{2}$ was multiplicated by $D = 1.7$ to adjust for the rate of homozygosity obtained from the mean diagonal elements of ${\varvec{G}}$**,** $n_{s} = 3$ denote the number of replicates in the rhizobox experiment, and $\overline{{n_{ij} }}$ is the mean number of seeds that germinated per rhizobox. Heritability of average field records was calculated as:

7 $$h_{a}^{2} = \frac{{D \sigma_{a}^{2} }}{{D \sigma_{a}^{2} + \frac{{\sigma_{a\gamma }^{2} }}{{n_{L} }} + \frac{{\sigma_{{\beta_{\tau } }}^{2} }}{{n_{C} }} + \frac{{\sigma_{\epsilon}^{2} }}{{n_{R} }} + \frac{{\sigma_{e}^{2} }}{{n_{T} }}}}$$

where $n_{L} = 3$ is the number of locations per family, $n_{C} = 21.6$ is the mean number of observations per trial-cut, $n_{R} = 4$ is the total number of replicates per family across all locations and $n_{T} = 9.8$ is the mean total number of cuts for each plot. For calculation of the individual heritability in the three locations the following numbers were used, $n_{L} = 1$, $n_{R} = 2$ in DK and $n_{R} = 1$ in UK and IR.

The bivariate model describes the additive effects in boxes and the additive effects in fields, and the genetic variations of the two traits are described by the bivariate Gaussian distributions of breeding values in $\left( {\alpha_{i} ,a_{i} } \right)^{{\text{T}}}$ (Falconer and Mackay 1996). In such a bivariate distribution the correlated effect on $a_{i}$ from $\alpha_{i}$ is obtained by

8 $$a_{i} |\alpha_{i} = b\alpha_{i} = \frac{{\sigma_{\alpha a}^{{}} }}{{\sigma_{\alpha }^{2} }}\alpha_{i}$$

where $b$ is the regression coefficient, $\sigma_{\alpha }^{2}$ and $\sigma_{\alpha a}^{{}}$ are genetic variance and genetic co-variance described in the bivariate model above Eq. 3.

Different genetic potential was assumed for the lines in the three locations and thus model with four dimensions was introduced, and thereby the correlated effect from $\alpha_{i}$ on $a_{li}$ is obtained by

9 $$a_{li} |\alpha_{i} = b_{l} \alpha_{i} = \frac{{\sigma_{\alpha al} }}{{\sigma_{\alpha }^{2} }}\alpha_{i}$$

where $b_{l}$ is the regression coefficient associate to the location $l = \left\{ {1,2,3} \right\}$ and $\sigma_{\alpha al}^{{}}$ are genetic co-variance described in Eq. (4).

All statistical analyses and model fitting described above were conducted using the DMU software package (Madsen and Jensen 2013).

### Response to selection

According to Falconer and Mackay (Falconer and Mackay 1996) the genetic response to selection per generation, $R$, is given by the equation:

10 $$R = ir\sigma_{a}$$

where $i$ is the selection intensity, $r$ is the accuracy, $\sigma_{a}$ the genetic standard variance of yield. In the present cases, we aimed to estimate the response to selection for two traits using combined data from both rhizoboxes and field trials. This introduces a multivariate selection approach (Mrode 2014), represented as:

11 $$\sigma_{I}^{2} = {\mathbf{g}}^{{\varvec{T}}} {\mathbf{P}}^{ - 1} {\mathbf{g}}$$

where $\sigma_{I}^{2}$ is the variance of selection index for the combined selection information from rhizoboxes and field trails, ${\mathbf{g}} = \left( {\sigma_{\alpha }^{2} ,\sigma_{\alpha a} } \right)^{{\varvec{T}}}$ is the vector of genetic variances and co-variances described in Eq. 3, and ${\mathbf{P}}$ is the phenotypic covariance matrix for the two traits. The diagonal values in ${\mathbf{P}}$ were obtained similarly to the denominator values used for calculating narrow sense heritability in Eqs. (6) and (7) and the off-diagonal elements in ${\mathbf{P}}$ were identical to $\sigma_{\alpha a}$. The accuracy of the combined selection information is given by $r = \sigma_{I} /\sigma_{a}$ and the response to selection is $R = i\sigma_{I}$ (Mrode 2014). All calculations described above were performed in R R Core Team (2023).

## Results

### Greenhouse and field data

After 21 days under greenhouse conditions, the mean number of germinated seeds per rhizobox was 2.7 (SD 0.5), with a mean TRL of 3.19 kilo pixels, and mean RA of 52.3 degrees. Both traits showed high phenotypic variation, with coefficient of variation (CV%) of 40.4 for TRL and 34.3 for RA (Table 1).

**Table 1:** Summary of the observed traits for plants grown under greenhouse conditions and field conditions for the 239 families. Root length and angle were recorded in greenhouse and yield of each cut was recorded in the field trials.

	No. of obs	No. of cuts	Mean	Std. dev	CV%
Total root length (kpx)	700	–	3.19	1.29	40.39
Seminal root angle (deg)	668	–	52.3	18.0	34.30
Yield, all cuts (kg)	9190	32	11.24	11.15	99.20
Yield (kg), year 1, DK	1888	4	18.83	15.4	81.80
Yield (kg), year 1, UK	928	4	20.07	16.61	82.77
Yield (kg), year 1, IE	2094	9	6.67	2.68	40.18
Yield (kg), year 2, DK	1887	4	9.88	6.65	67.32
Yield (kg), year 2, UK	1010	5	7.52	7.53	100.1
Yield (kg), year 2, IE	1383	6	6.47	2.76	42.68

*DK *  Denmark, *UK*  United Kingdom, *IE*  Ireland, year 1 indicate the first year of harvests (2021) and year 2 the second (2022)

**Fig. 1:**
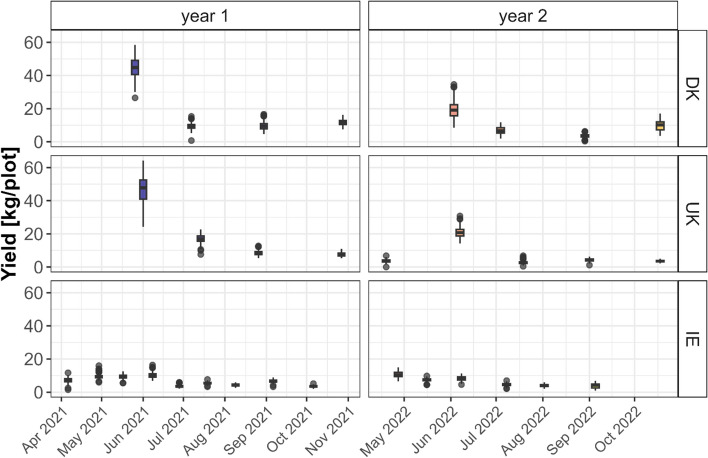
Yield recordings (n = 9190) for each location (Denmark, UK, and Ireland) and cut across two growing seasons of the experiment

A total of 9,190 yield recordings were collected across the two years of field trials at the three locations. Ireland recorded a higher number of cuts per season (up to nine), resulting in more yield measurements per family and contributing to the phenotypic variation observed. Yield values ranged from below 5 kg to 60 kg per plot (Fig. 1), with a CV% of 99.2 across all cuts and locations, reflecting the substantial variability due to environmental conditions and management practices. To account for this variation, yield data were log-transformed. Figure 1 shows yield patterns across locations, years, and seasons, reflecting the impact of management practices. In Denmark and the UK, the first cuts of the season in early spring were significantly larger than subsequent cuts, while cuts in Ireland showed more uniform yields across the growing season.

### Covariance estimates

Variance components were estimated using bivariate models for TRL and field yield, as well as for RA and field yield, assuming identical genetic variation across locations (Eq. 3). The variance of the family-location interaction effect (${\sigma }_{a\gamma }^{2}),$ was small, with values of 0.0003 for TRL and $0.0004$ for RA. However, when models were fitted with location-specific genetic effects (Eq. 4), the family-location interaction effect became negligible, i.e., ${\sigma }_{a\gamma }^{2}=0.000$ (Table 2). This indicates that environmental factors played a larger role in yield variation than genetic differences between locations. In both bivariate models, the variance components for the combined field trial and cut effects (${\sigma }_{\beta \tau }^{2}$) were significant, with values of 0.0293 for the model with TRL and 0.0300 for the model with RA. The associated standard errors were 0.0022 and 0.0023, respectively. (Table 2).

**Table 2:** Estimated residual variances of TRL and RA records in greenhouse, and variances of environmental and genotype by location effects, i.e., trial-cut, family-location, plot, and residual effects of yield recorded in the field experiment in three locations: Denmark, UK, and Ireland. The model includes two genetic effects: **α**, the family-level additive genetic effect for greenhouse root traits, and **α**, the additive genetic effect for field yield. Location-specific genetic effects denoted by **a₁**, **a₂**, **a₃** for Denmark, UK, and Ireland, respectively. Variances represent different sources of variation, including genetic and environmental components. Standard errors are shown in parentheses

Traits	Variance component	Estimates of model with $\boldsymbol{\alpha }$ and ${\varvec{a}}$	Estimates of model with $\boldsymbol{\alpha }$, ${{\varvec{a}}}_{1}$, ${{\varvec{a}}}_{2}$, and ${{\varvec{a}}}_{3}$
Field yield (kg)*	Trial-cut, $\sigma_{\beta \tau }^{2}$	0.030 (0.002)	0.029 (0.002)
	Family-location, $\sigma_{a\gamma }^{2}$	0.0003 (0.0003)	0.0000 (0.0004)
	Plot, $\sigma_{@}^{2}$	0.0016 (0.0003)	0.0010 (0.0003)
Root length (kpx)	Residuals, $$\left\{ {\begin{array}{*{20}c} {{ }\sigma_{\varepsilon }^{2} } \\ {\sigma_{e}^{2} } \\ \end{array} } \right.$$	$2.12{ }\left( {0.13} \right)$	$2.12 \left( {0.13} \right)$
Field yield (kg)*		$0.0196{ }\left( {0.0003} \right)$	$0.0196{ }\left( {0.0003} \right)$
Field yield (kg)*	Trial-cut, $\sigma_{\beta \tau }^{2}$	0.030 (0.002)	0.029 (0.002)
	Family-location, $\sigma_{a\gamma }^{2}$	0.0004 (0.0003)	0.0000 (0.0004)
	Plot, $\sigma_{@}^{2}$	0.0016 (0.0003)	0.0010 (0.0003)
Root angle (deg)	Residuals, $$\left\{ {\begin{array}{*{20}c} {{ }\sigma_{\varepsilon }^{2} } \\ {\sigma_{e}^{2} } \\ \end{array} } \right.$$	697.7 (46.3)	695.1 (46.0)
			0.0196 (0.0003)
		0.0197 (0.0003)	
Field yield (kg)*			

*For field yield, the log-transformed values were used to adjust for the inhomogeneity of variation in yield at the three locations in Denmark, UK, and Ireland

### Genetic correlations

The genetic correlation between greenhouse-recorded root traits and yield from field cuts observed across the three locations was 0.40 for TRL and 0.15 for RA (Table 3). The correlation of 0.40 (SE = 0.14) between TRL and yield across all cuts, locations and years indicates a significant shared genetic influence. In contrast, the correlation of 0.15 between RA and yield, although positive, had a relatively high standard error of 0.14, suggesting a weak and uncertain association.

**Table 3:**
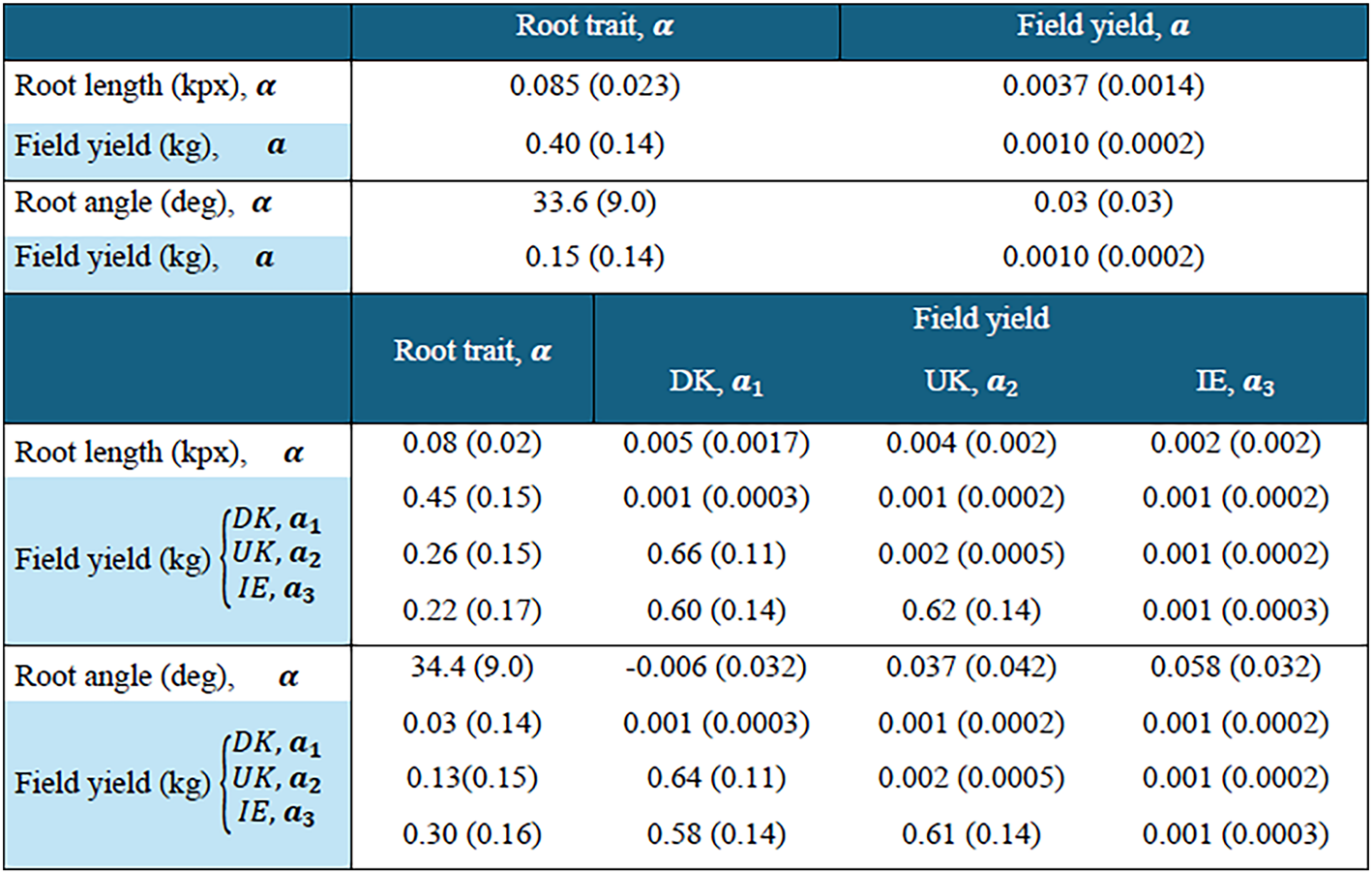
Estimated variances (in diagonal), co-variances (above diagonal) and correlations (below diagonal) of the additive genetic effects of root length and angle recorded in greenhouse (**α**) and yield recorded in the field experiment in three locations (**a**_**1**_, **a**_**2**_, **a**_**3,**_): Denmark, UK, and Ireland. Standard errors are shown in brackets

**Fig. 2:**
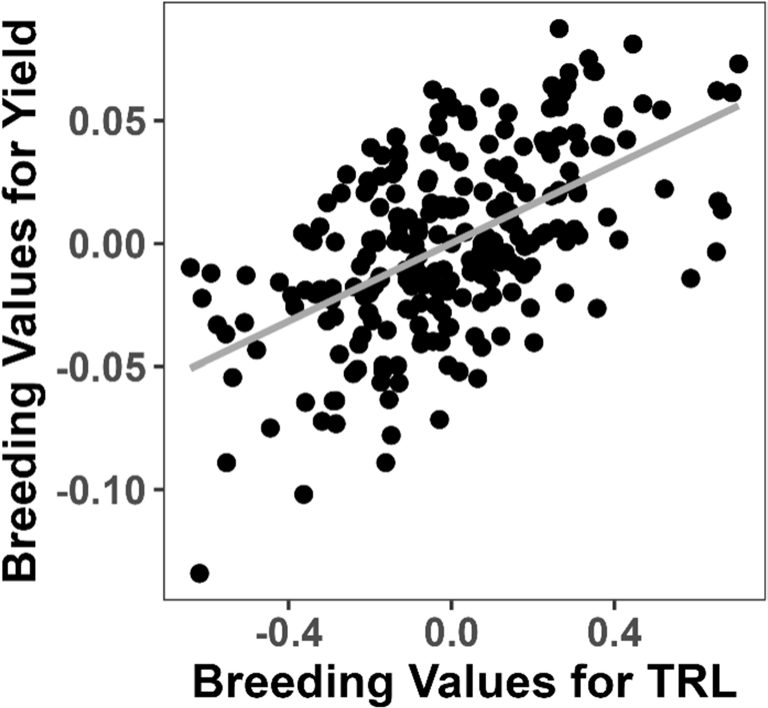
Scatter plot showing the genetic association between predicted breeding values for total root length (TRL) measured in greenhouse rhizobox experiments and field yield across all locations and years. Each point represents one of the 239 families evaluated. The x-axis displays the breeding values for TRL (kilopixels), and the y-axis shows the breeding values for log-transformed field yield (kg per cut). The solid line represents the linear regression fit, indicating a positive association between TRL and yield. The genetic correlation between TRL and field yield was estimated to be 0.40 (standard error = 0.14)

These genetic correlations indicate that breeding values for TRL and yield are moderately related (Fig. 2). To quantify this relationship further, we calculated the genetic regression coefficient *b* using Eq. (8) $b = \frac{{\sigma_{\alpha a}^{{}} }}{{\sigma_{\alpha }^{2} }} = \frac{0.0037}{{0.085}} = 0.044$ where $\sigma_{\alpha a}^{ }$ is the genetic covariance between TRL and yield and $\sigma_{\alpha }^{2}$ is genetic variance of TRL (as per Eq. (8). This coefficient suggests that an increase in TRL by one kilopixel in the greenhouse corresponds to a proportional increase of $e^{0.044} = 1.045$ or 4.5%, in field yield, considering the log-transformed response variable.

Figure 2 illustrates the genetic association between the predicted breeding values for TRL and field yield. The breeding values for TRL ranged from –0.64 to 0.70 kilopixels, while those for the log-transformed field yield ranged from –0.13 to 0.09 kg per cut. The positive slope observed in Fig. 2 reflects the genetic correlation of 0.40, demonstrating that genotypes with higher breeding values for TRL tend to also have higher breeding values for yield. Genetic correlations between TRL and field yield were also estimated separately for each location using models with location-specific genetic effects. The correlations were 0.45 (SE = 0.15) in Denmark, 0.26 (SE = 0.15) in the UK, and 0.22 (SE = 0.17) in Ireland (Table 3). These results demonstrate a consistent positive relationship between TRL and yield across different environments, although the strength of the association varies by location. In contrast, the genetic correlations between RA and field yield were more variable and generally weaker:  – 0.03 (SE = 0.14) in Denmark, 0.13 (SE = 0.15) in the UK, and 0.30 (SE = 0.16) in Ireland suggesting a less consistent relationship.

### Location-specific genetic correlations

**Fig. 3:**
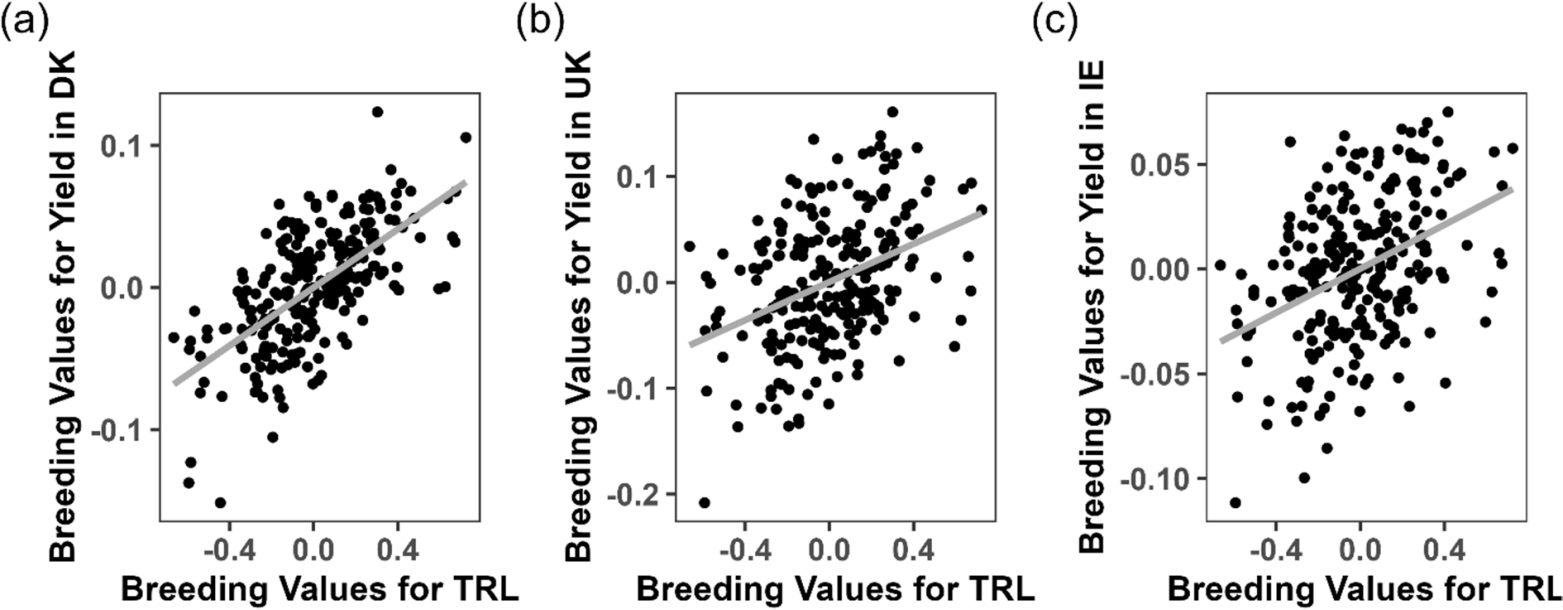
Scatter plots showing the genetic associations between predicted breeding values for TRL measured in greenhouse rhizobox experiments and field yield at each of the three locations: **a** Denmark, **b** United Kingdom, and (**c**) Ireland. Each point represents one of the 239 families evaluated. The x-axis displays breeding values for TRL (kilopixels), and the y-axis shows breeding values for log-transformed field yield (kg per cut). Solid lines represent linear regression fits for each location. The genetic correlations between TRL and field yield were estimated to be **a** 0.45 (standard error = 0.15) for Denmark, (**b**) 0.26 (SE = 0.15) for the UK, and **c** 0.22 (SE = 0.17) for Ireland

Genetic correlations between TRL and field yield in the three locations are based on estimated covariances of 0.005 (Denmark), 0.004 (UK), and 0.002 (Ireland), along with their associated variances. Using Eq. (9), the coefficients of the regression lines associated with the plots of breeding values of TRL in the greenhouse and field yield in the three locations were calculated as: $b_{DK} = \frac{{\sigma_{\alpha aDK}^{{}} }}{{\sigma_{\alpha }^{2} }} = \frac{0.005}{{0.08}} = 0.06$, $b_{UK} = \frac{0.004}{{0.08}} = 0.05$, $b_{IE} = \frac{0.002}{{0.08}} = 0.025$.. Since log-transformation of yield was used as the response, the associated proportion of yield increase for each location was given by $e^{bl} :$ $e^{0.06} = 1.064$ or 6.4% increase in Denmark, $e^{0.05} = 1.051$ 5.1% increase in UK and $e^{0.025} = 1.025$ or 2.5% increase in Ireland. These results indicate that TRL measurements from the greenhouse can predict deviations and genetic progress for yield in these locations. Specifically, genetic progress could be twice as high in Denmark compared to Ireland for the same selected families when selected based on the same rhizobox records (Fig. 3).

### Heritability estimates

Narrow-sense heritabilities (h^2^) of the analyzed traits were calculated using the variance components estimated from the bivariate models described in Eq. 3 and 4. Heritabilities of root traits were calculated using Eq. (6), and of field yield with Eq. (7). Using the bivariate model assuming identical genetic variances across locations (Eq. (3)), the heritability of TRL was estimated to be 0.36, and RA was 0.39 (Table 4). The heritability of field yield across all locations, years, and cuts was estimated to be 0.31. When using the bivariate model with location-specific genetic variances (Eq. (4)), the heritability estimates for TRL and RA were 0.35 and 0.40, respectively (Table 4). The heritability estimates for field yield varied by location: 0.39 for Denmark, 0.48 for the UK, and 0.30 for Ireland.

**Table 4:** Narrow sense genetic heritability (h^2^) estimates for yield, total root length and root angle, based on two types of bivariate models: one assuming a single identical genetic effect across locations (**α** and **a**) and another allowing for location-specific genetic effects (**α**, **a**_**1**_, **a**_**2**_, and **a**_**3**_) in Denmark, UK, and Ireland. To standardise the variance across the three locations, yield values were log-transformed

Traits	Estimates of model with $\boldsymbol{\alpha }$ and ${\varvec{a}}$	Estimates of model with $\boldsymbol{\alpha }$, ${{\varvec{a}}}_{1}$, ${{\varvec{a}}}_{2}$, and ${{\varvec{a}}}_{3}$
Root length (kpx)	0.36	0.35
Field yield (kg)	0.31	0.39, 0.48, 0.30
Root angle (deg)	0.39	0.40
Field yield (kg)	0.31	0.39, 0.48, 0.30

### Response to selection

The benefit for commercial breeding was evaluated through two scenarios. The first scenario considered a breeding program relying solely on field recordings, while the second scenario incorporated additional data from three rhizoboxes per family, similar to the present experiment. In scenario 1, we calculated the expected genetic gain from the current field experiment involving 239 F_2_ families and 4 replicates. Assuming that 2 families are selected as the final outcome, the proportion of selection is 2/239, and the selection intensity is $i=2.7$. In scenario 2, we performed pre-selection using rhizobox data, doubling the number of F_2_ families to 478. From these 478 families, we selected the top-performing half (239 families) for further field trials, based on root development traits in the rhizoboxes. This increased the selection intensity, resulting in a proportion of selection of 2/478 and a corresponding selection intensity of $i=2.9$.

**Fig. 4:**
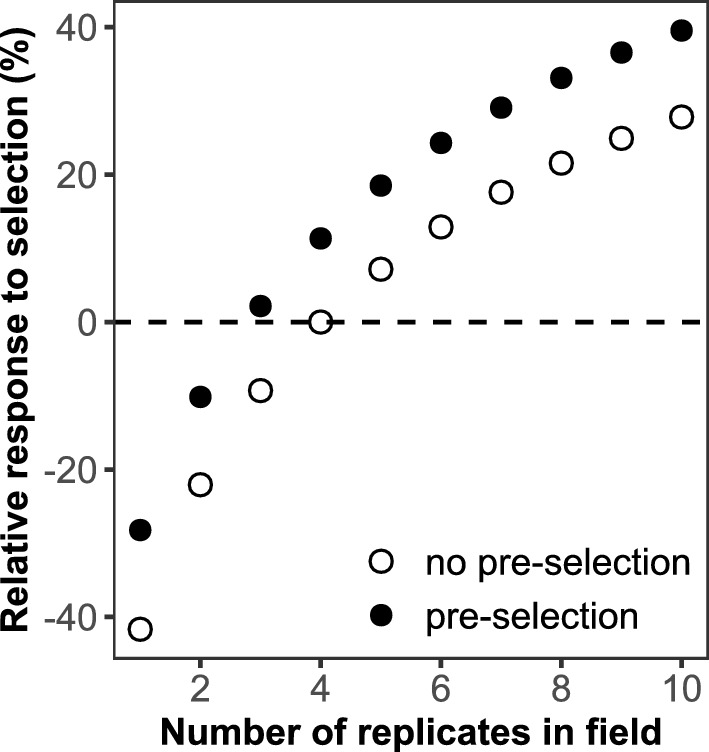
The expected genetic gain in two breeding scenarios: one without pre-selection from rhizoboxes (field only, empty black circles) and another incorporating pre-selecting data from three rhizoboxes per family (solid black points). The genetic gain is presented relative to the baseline genetic gain obtained from a field experiment with four replicates and no rhizobox information

The expected response to selection was calculated using Eq. (10) and the results are summarized in Fig. 4. The calculations considered varying numbers of field replicates, ranging from 1 to 10. The expected genetic gain is presented relative to a baseline genetic gain obtained from scenario 1, assuming 4 field replicates without rhizobox information. Both scenarios showed that genetic gain increased with the number of field replicates. Furthermore, incorporating rhizobox information added approximately 10% more genetic gain across all levels of field replication.

## Discussion

Our study focused on early root development traits to increase the genetic gain in biomass yield of perennial ryegrass. We examined the heritability and genetic correlations of these traits across multiple years and locations in a perennial ryegrass breeding program. We hypothesized that early root traits measured in controlled greenhouse environments could serve as an indicator of long-term field yield. Our findings demonstrated a positive genetic correlation between traits like TRL in 21-day-old seedlings and field yield. These results support integrating root trait measurements into breeding strategies to promote the development of high-yielding perennial ryegrass varieties.

The narrow-sense heritability of yield in perennial ryegrass varies considerably, due to factors such as population, location, year, and cutting practices (Fè et al. 2015b; Fernando et al. 1997; Ravel and Charmet 1996). In our study, conducted over different years and locations, we observed a moderate narrow-sense heritability for yield at 0.31. These findings align well with previously reported heritability estimates across different environments, which typically range between 0.30 and 0.35 (Fè et al. 2015b; Jahufer et al. 2021; Lin et al. 2017) and within locations within specific environments, which show a broader range of heritability estimates from 0.44 to 0.60 (Fè et al. 2015b; Guo et al. 2018).

Similarly, when examining root traits, our study identified a heritability of 0.36 for TRL of 21-day-old seedlings measured in rhizoboxes, which closely matches previous reports of a heritability of approximately 0.35 for total root weight in mature plants grown in tubes (Crush et al. 2006). This consistency across different developmental stages and methodologies suggests that early-stage TRL measurements may serve as a reliable proxy for overall root biomass, providing a potentially practical approach for early selection in breeding programs (Malinowska et al. 2017; Tackenberg 2006).

For RA, we observed a narrow-sense heritability of 0.39, which is comparable to that of TRL. However, this contrasts with significantly higher broad-sense heritability reported for seminal root angle in other crops such as rice (Uga et al. 2015), wheat (Colombo et al. 2022; Richard et al. 2015) and barley (Jia et al. 2019). These differences between narrow and broad-sense heritability suggest that the genetic regulation of root angle is more complex, potentially involving significant non-additive genetic effects. This complexity underscores the need for further research into seminal root angle to clarify the underlying genetic mechanisms.

Examining the genetic correlations between root traits and yield can help us understand how root architecture might influence crop productivity (Ogrodowicz et al. 2023; Robinson et al. 2018). In our study, the genetic correlations between TRL and yield across all cuts, years, and locations were moderately strong and positive (0.40). This consistent positive correlation, observed over two growing seasons, suggests potential benefits of early root system development for sustained yield (Chesworth et al. 1998; Viana et al. 2022). This persistence may be explained by the influence of locally (or broadly) adapted alleles, as demonstrated by Chen et al. (Chen et al. 2021) in switchgrass, who found that these alleles contribute to the positive genetic correlation between above- and belowground biomass, influencing both shoot and root development. Similar findings have been reported in grain crops, where specific root traits observed under controlled glasshouse conditions in barley (Robinson et al. 2018) and wheat (Bai et al. 2019; Xie et al. 2017), were correlated with grain yield, highlighting the broader role of root architecture in resource acquisition and productivity.

In addition to genetic correlations, the dynamic interaction between root and shoot growth is important for maintaining plant productivity under stress conditions. Li et al. (Li et al. 2021a, b), demonstrated that reduction in root biomass due to defoliation led to decreased shoot growth in perennial grasses, highlighting the importance of root plasticity and shoot–root interactions in maintaining plant performance. This supports the idea that robust early root development not only enhances root growth but also contributes to shoot productivity through coordinated genetic and physiological feedback mechanisms, underscoring the value of root traits in improving yield outcomes.

While our observed genetic correlations indicate a relationship between root traits and yield, these relationships are not definitive. Other genetic factors, independent of the traits measured, may also play a role in determining yield outcomes. The bivariate model used to assess yield variation (Table 2) captures only a portion of the variance, suggesting that other factors—such as environmental variability, genotype-by-environment interactions, or unmeasured genetic traits—may also significantly impact yield. In addition to genetic factors, robust root development and turnover may enhance soil water retention, particularly in water-limited environments, thus indirectly contributing to improved yield (Macleod et al. 2013). Therefore, exploring the specific genetic correlation between root traits and yield across diverse environmental conditions can be useful for refining location-specific breeding strategies aimed at improving yield stability. The three experimental locations—Denmark, Ireland and UK—fall within the Atlantic maritime macroclimate of Northwestern Europe (Bednar-Friedl et al. 2022). Despite this general climatic similarity, variations in microclimate, local soil types (Joint Research Centre, European Soil Data Centre 2024), and cut management practices, ranging from about four cuts per year in UK and Denmark to nine in Ireland, influenced our results (Fig. 1). The bivariate model showed varying degrees of genetic correlation between the TRL and biomass yield in each location. Although all locations had a positive genetic relationship between these traits, the strength varied significantly, with correlations in UK (0.25 + − 0.15 SE) and Ireland (0.22 +  − 0.17 SE) being nearly half of those observed in Denmark (0.45 and 0.15 SE). Such inconsistencies in the relationship between root traits and agronomic performance have also been reported in other species (Colombo et al. 2022; Robinson et al. 2018), suggesting that the adaptive value of early root development depends on specific environmental conditions.

Root angle is an important trait for understanding plant performance and yield potential. While studies have demonstrated the influence of RA on grain yield in various crops, its specific impact on biomass yield in perennial forage crops remains less explored (Fusi et al. 2022; Uga et al. 2013). In our study, we found a positive genetic correlation between RA and field yield (0.15), although the substantial standard error (0.14), suggests that this result should only be considered indicative (Table 3). Further analysis of the genetic correlations between RA and yield across the three locations revealed distinct patterns compared to TRL. In Denmark and UK, correlations between RA and yield were near zero or non-significant, with high standard errors. In contrast, Ireland showed a positive correlation (0.30, 0.16 SE) between RA and yield, while the correlation for TRL was lower in comparison to the other locations (Table 3). These findings point to a potential location-specific effect of RA on yield, indicating that different types of root architecture may provide growth advantages under varying environmental conditions (Hostetler et al. 2024; Lynch 2021; Rogers and Benfey 2015).

Perennial ryegrass demonstrated heritable variation in both above- and below-ground traits (Table 4), indicating a substantial genetic basis for their development. Nonetheless, both shoots and roots are highly influenced by environmental factors, dynamically adapting to various stressors and biological interactions (Rich and Watt 2013; Li et al. 2021a, b). This interaction between genetic and environmental influences is highlighted by the genetic correlations observed in our study, which vary for different locations. Despite occupying distinct environments and serving different functions, the genetic correlation between shoot biomass and below-ground traits suggests that shared genetic mechanisms may influence their coordinated development and growth. This is consistent with other studies that have suggested coordinated regulation through shared genetic pathways or interconnected biological processes (Puig et al. 2012; Naz et al. 2013; Siddiqui et al. 2020; Chen et al. 2021; Li et al. 2021a, b).

Incorporating early root traits into selection can enhance genetic gain in biomass yield. Our comparison of breeding scenarios indicates that pre-selection using rhizobox data increases selection intensity and genetic gain by approximately 10% compared to field-only methods (Fig. 4). By measuring root traits in young seedlings under controlled conditions, we can pre-select the most promising families before sufficient seeds are available for field testing. This early selection reduces the number of families that need to be evaluated in the field, directly addressing the limitations of traditional field trials—which are often constrained by space, time, and cost—while allowing for a larger number of entries and replicates. While only nine seeds per family were scored for TRL and RA, heritability estimates and significant genetic correlations with field yield indicate that these samples sufficiently captured between-family genetic variation. Root traits measured on these nine plants were also correlated with biomass yield data collected from a much larger population in the field, further supporting the robustness of our findings. Similar strategies have been explored in other crops, such as wheat, where integrating root traits like deeper root length has shown potential for improving breeding efficiency, yield, and stress tolerance (Tracy et al. 2020; Ober et al. 2021). However, further research is needed to determine the optimal number of families for pre-selection to ensure efficiency.

Our study highlights the value of early root development traits, particularly TRL, in enhancing genetic gain in perennial ryegrass breeding programs. We hypothesized that root traits measured in young seedlings could act as indicators for field yield across several environments and years, and our results confirmed this hypothesis. TRL showed significant heritability and a positive genetic correlation with field yield, suggesting that incorporating early root traits into selection criteria can improve yield outcomes.

Incorporating early root traits alongside conventional field traits can increase genetic gain by improving prediction accuracy and selection intensity, ultimately contributing to the development of high-yielding ryegrass varieties for future biomass production.

## Supplementary Information

Below is the link to the electronic supplementary material.

Supplementary file1 (PDF 447 KB)

## Data Availability

We make our datasets freely available under Creative Common license at https://zenodo.org/records/14499394 and https://github.com/martamalinowska/rhizobox-field.
