# Supplementary material for: The value of early root development traits in breeding programs for biomass yield in perennial ryegrass (Lolium perenne L.)
Source: Theor Appl Genet. 2025 Jan 21;138(1):31. doi: 10.1007/s00122-024-04797-5 (PMC11750904; doi:10.1007/s00122-024-04797-5)
Supplement: Supplementary file 1 — Supplementary file1 (PDF 447 KB) [file 122_2024_4797_MOESM1_ESM.pdf]

Supplementary Information

The Value of Early Root Development Traits in Breeding Programs for Biomass Yield in Perennial Ryegrass (*Lolium perenne* L.)

Malinowska, M[1*].; Kristensen, P.S[1].; Nielsen, B[.1;] Fè, D[.2;] Ruud, A.K[.1,3]; Lenk, I[.2]; Greve, M[.2;] Asp T.[1]

Supplementary Figure 1. (a) Rhizoboxes arranged in stands within the greenhouse, used for phenotyping root traits in perennial ryegrass. (b) Close-up image of 21-day-old seedlings growing in a rhizobox

(a)

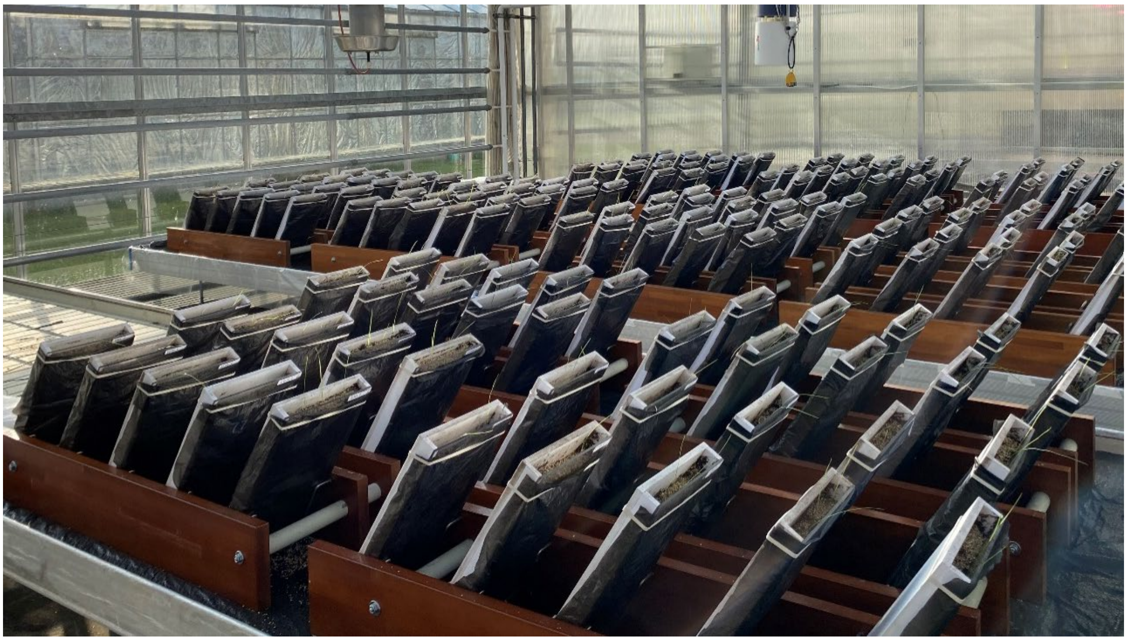

(b)

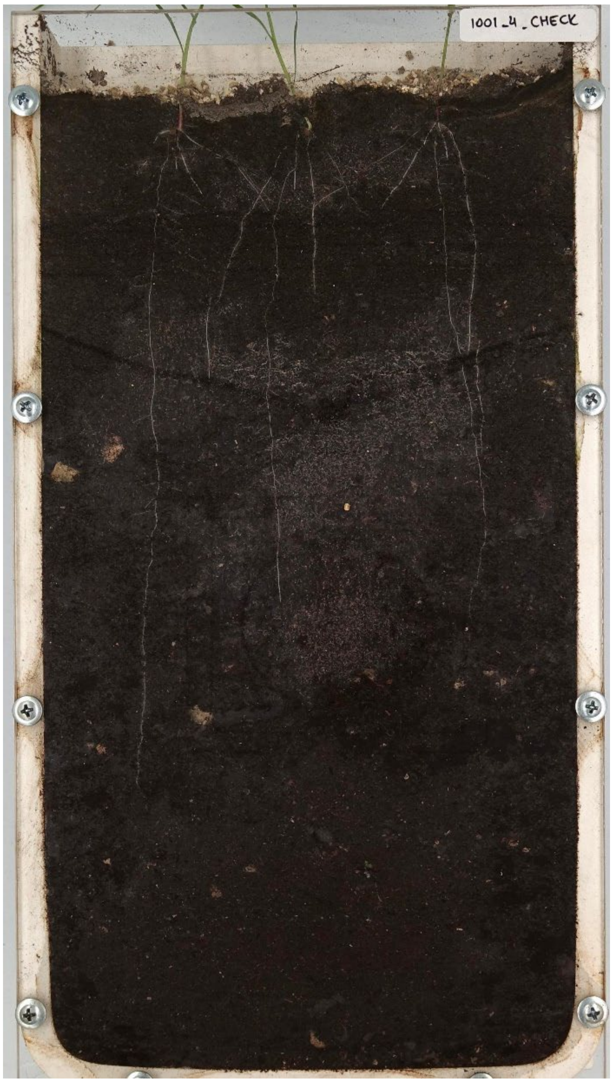
1001_4_CHECK
